# The impact of centralization on structural changes in healthcare: when it works

**DOI:** 10.3389/frhs.2025.1484225

**Published:** 2025-02-10

**Authors:** Sergey Shishkin, Igor Sheiman, Elena Potapchik, Vasily Vlassov, Svetlana Sazhina

**Affiliations:** National Research University Higher School of Economics, Moscow, Russia

**Keywords:** healthcare governance, centralization, structural changes, healthcare system, health governance

## Abstract

**Purpose:**

After a decade of post-Soviet decentralization of the healthcare in Russia the opposite trend has been dominating. This paper explores the impact of centralization of healthcare governance on the structure of the healthcare system in Russia, including shift in service delivery structure, the institutional organization of healthcare providers, and their interactions.

**Methodology:**

We employ quantitative and qualitative analysis to study how centralization has contributed to restructuring service delivery with instruments of utilization planning, vertical health programs, and centrally determined pathways of patients flows in a multi-level health care system.

**Findings:**

Centralization of healthcare governance has contributed to restructuring the Russian healthcare system, providing positive changes in structure of inpatient/outpatient care utilization, the organizational structure of service providers, and the structure of their activities. Inpatient care is increasingly replaced by outpatient care and day wards. Centralization contributed to creation of new types of medical organizations (perinatal centers, vascular centers, etc.), development of prevention, and strengthening of providers activity integration in a multi-level system of medical care. However, centralization has not been accompanied by the effective interaction of different levels of governance in developing structural reforms and their implementation in the regions. Uniform activities for the entire country do not take into account specific regional and local conditions. Some unified solutions are implemented in regions with negative consequences for the accessibility of care locally. The excessively centralized model of preventive measures does not provide an effective balance between detection of diseases and follow-up treatment. A so-called “new primary healthcare model”, initiated from the top, limits the development of alternative models that are needed in many regions of the country. The analysis concludes with a set of conditions that should be followed in designing and implementing a centralized model of healthcare governance.

## 1 Introduction

The search for the better healthcare governance model has been initiated in many countries over the last decades with the focus on decentralization. This has become a common area of innovation to improve the performance of healthcare systems. Decentralization in healthcare is usually considered as the “downwards” transfer of formal responsibility and power to make decisions regarding the management, production, distribution and/or financing of health services from national government to subnational governments (either regional or local). The reverse “upwards” transfer of competencies and prerogatives is centralization (1).

The reviews of studies examining the impact of decentralization on outcomes and health system performance show that decentralization is associated with improvements in selected healthcare outputs and health outcomes (2–4). Despite some positive results of decentralization, it created problems, the solution of which in a number of countries required certain steps to centralize health care governance: increasing interregional inequality in the availability of medical care, disruption of coordination of regional and central organizations, and others. In countries where historically there were decentralized models of healthcare governance, these problems were solved by the development of common standards of interaction, common information mechanisms (Spain) (5, 6), the creation of national systems for coordination and quality assurance (Switzerland) (7), and strengthening a national center to coordinate the activities of regional systems (Portugal) (8). Norway has taken the path of centralizing tertiary care (9). In countries with an initially centralized system, decentralization attempts were then replaced by a tendency to restore certain functions of the national center [Italy (10, 11), Brazil (12–14), New Zealand (15)]. In Latvia, initial post-Soviet decentralization was unsuccessful, and the country returned to a centralized national system (16).

There are theoretical and evidence-based arguments favoring centralization. Organizational theory provides arguments that centralization can: (1) provide clearer steering signals; (2) facilitate standardization of processes and products; and thus (3) improve predictability in organizational practice (17, 18). Public administration theory includes arguments that centralization can provide better possibilities for setting standards and holding delivery organizations accountable to uniform principles. It may also strengthen the capacity for planning and coordinating service levels across the system. Medical technology developments in some instances point to a need for centralization in order to support a higher degree of specialization. This can, for example, be seen in the case of sophisticated scanners, which are expensive and cannot be purchased for all providers in local areas (19).

However, centralization may lead to weakening the responsibility of regional and local authorities for the accessibility and efficiency of healthcare. In contrast, decentralization provides a stronger link between decision-makers and users of services and can therefore ensure better accountability of the government. Decentralized decision-making facilitates the use of knowledge and experience accumulated by local staff, and can improve the flexibility and adaptability of the system (19). There is a clear risk of over-investment and poor or inappropriate use if decision making is decentralized without a proper coordination mechanism. Coordination problems in decentralized systems and the risk of duplication of services are major arguments for centralizing some degree of power (20).

The claims of performance benefits related to centralization vs. decentralization are not universal. The outcomes depend on the specific historical and ideological context. Correspondingly, centralization/decentralization processes represent recurring cycle, and the related decisions need to be regularly revisited and re-adjusted (21).

Studies of centralization/decentralization in healthcare explore mostly their impact on public health, quality and accessibility of care (22–24). Less represented are studies of the impact on structural changes in healthcare systems, including shifts in the ratio of volume of the healthcare sectors and the organizational structure of service delivery.

The subject of this study is Russia's experience over the last 30 years. Russia is a large country with a three-level centralized governance system: federal–regional–municipal. Regions vary substantially in population from around 50,000 in Chukotsky region to 13 million in Moscow, with a third of regions having populations of more than 2 million. The significant size of the country and the heterogeneity of regions determine the need to build a governance system that can take into account the advantages and disadvantages of centralization and decentralization. The pattern of healthcare governance has changed radically over the last three decades in the opposite directions –first decentralization, then back to centralization.

The healthcare system of Russia was inherited from the Union of Soviet Socialist Republics (USSR) with its' serious structural problems. The main ones were the dominance of hospitals, the weak primary healthcare (PHC), insufficient differentiation of hospitals in terms of the intensity of medical care and the composition of patients, imbalances inhuman resources, including an excess of hospital doctors and a shortage of PHC doctors, insufficient and inequal funding (25).

In the early 1990s, in the process of building the federative state in Russia the centralized governance of health care was dismantled and replaced by a decentralized system. It contributed to the fragmentation of the healthcare system and the inequality in the accessibility of care across regions and municipalities. Priority in the allocation of resources was given to hospitals; outpatient care was financed on a residual basis. The gatekeeping function of PHC providers weakened, which contributed to the shortage of specialists in outpatient settings. The provision of preventive services was significantly reduced (26–28). In the 2000s Russian state began to pursue a policy of centralizing public administration. The aim has been to implement effective vertical executive control, and reintegrate the Russian legal' space.

This study assesses the impact of the centralization of healthcare governance on structural changes in the Russian healthcare system. We focus on five dimensions of structural changes: (1) the proportions of volumes of various types of medical care; (2) the organizational structure of service providers; (3) the structure of their activities; (4) the ways they interact with each other; and (5) the structure оf mandatory health insurance (MHI) funds. We consider also the impact of centralization on the accountability of health authorities, on disparities in access to free medical care, and on health outcomes. The paper has the following organization. At the first step, we provided a general description of the Russian centralization policy in the last two decades, including the identification of management tools used to enforce structural changes.

Second, we analyzed the structural changes which were a direct result of decisions made by the centralized governance system. To describe structural changes, we used quantitative and qualitative indicators.

Third, we described the problems caused by centralization.

Fourth, we summarized the achievements and problems of centralization, assessed the differences between Russian and international approaches to centralization, identified pre-conditions for the positive impact of centralization on structural changes, and formulated conclusions regarding further changes in healthcare governance.

## 2 Methods

The research method employed in the study can be classified as a combination of literature review, database search, content analysis.

To identify the main structural changes in healthcare during the last two decades we searched MEDLINE using the query: “Russia* AND “Delivery of Healthсare"[mh] AND (healthcare reform[mh] OR centrali* OR decentrali*) AND 1990:2023[dp]”. All 384 results were checked manually and 86 were relevant. We also searched the Russian Health сare database at the Central Medical Library. We searched for the Russian equivalents of Centralization, Decentralization, and of the 110 records found, 18 were relevant. We used sources snowballed from published reports. Also, we used the gray literature related to Russian healthcare, including those in limited circulation, unpublished documents, memorandums, and presentations from our personal collections covering study period. We used statistical data from the Russian sources—the Federal State Statistics Service and the Russian Research Institute of Healthcare Organization and Informatization. Some aspects of the structural transformations are covered using the data from interviews and polls of physicians we have done over the years.

To study the impact of centrally set health care utilization targets on the structure of service delivery, we collected data on the annual plans of utilization and compared them with the actual utilization data derived from the statistical sources. The analysis of the impact on the new organizational forms of service delivery was based on the study of the regulatory acts and the data on the implementation of the centralized plans in the regions of the country. Health finance centralization was explored with the use of statistical data of the Ministry of Finance and the Ministry of Health. Impact on the accountability of health authorities was examined by qualitative analysis of the regulatory acts.

To compare the international processes of centralization/decentralizations with the developments in Russia, we analyzed the studies of the WHO European Observatory on Health Systems and Health Reforms. The focus was the impact of these processes on the structure of service delivery.

To analyze regional disparities in access to healthcare, we used data of the survey “Readiness for Change”, conducted by the National Research University Higher School of Economics in 2021 (https://csils.hse.ru/monitoring_gkp). The sample represents the economically active population of the Russian Federation (from 15 to 72 years old) and is 6,000 respondents. Then the method of cross-tab analysis was used to determine the variation of care utilization and patients' satisfaction by regions and income groups. We also used surveys of medical workers to study primary health care, access to care, integration of providers. They have been published elsewhere with the detailed description of the methodology. The references to the corresponding publications are provided in the text. Health outcomes were presented with references to the sociological surveys and statistical data.

## 3 Results

### 3.1 General characteristics of health governance

In 1991, the vertical administrative subordination of healthcare authorities was replaced by a model of public administration based on the division of powers between federal government, regional governments and local authorities. The latter had a key role in healthcare. A limited range of management areas were listed where the decisions of federal authorities were binding for subordinates (setting standards and monitoring their compliance, licensing, sanitary and epidemiological surveillance, etc.). Outside of this set of areas, the healthcare system was to be governed primarily by regional and local governments. The mechanisms of interaction between government bodies, and their rights and responsibilities in resolving issues falling under joint jurisdiction were not clearly defined by law. In 1993, a decentralized MHI system was introduced.

Decentralization of governance was initially well received by the society as a part of the restructuring of the highly centralized Soviet state. The dominant public perception was that local health authorities would be better able to respond to the needs of the local population and ensure a more efficient allocation of resources. However, the management tools that the federal government retained were insufficient to effectively influence the policies of regional authorities. A similar situation was at the regional level in relations between regional governments and municipal authorities. Regional governments concentrated budgetary resources and MHI funds in the region and could use the economic dependence of financially weak municipalities for subsidies from the regional center. The power over financially secure municipalities (regional capitals and large cities) was very limited. The term “the municipalization of healthcare” appeared, meaning the deep decentralization of healthcare governance in the regions (27).

The consequences of decentralization did not meet the expectations. The economic capacity of most municipalities was not sufficient to build their own isolated systems and a culture of cooperation did not exist.

In the 2000s, a number of steps were taken to clarify the powers of federal, regional, and local health authorities. In 2006–2012, the powers of local authorities were almost completely transferred to regional governments. This was accompanied by the transfer of ownership of municipal service providers to the regions. A number of regulations were adopted aimed at expanding the power of the federal center over regions and their healthcare organizations, planning, and finance. The MHI system was reformed with a focus on the centralization of its funds and strengthening the central regulation of insurers (29).

Three major instruments are currently used by federal government to affect structure of the healthcare system:

• a federal program of state guarantees of free medical care to citizens;
• vertical federal programs;
• pathways of healthcare provision.

*The Federal Program of State Guarantees of free medical care to citizens* (PSG) is an annually updated federal document which defines the healthcare benefit package. In addition, PSG pursues two goals: establishing a balance between the volume of free medical care and the available amount of public funding and stimulating the structural changes in the healthcare system. The PSG sets healthcare utilization targets per capita (number of bed-days, physician visits, emergency care visits, etc.) and recommended unit costs. Regions are required to use these targets when developing their own territorial PSGs. If the federal target for the current year advices, for example, a reduction in the volume of inpatient care per resident, then regions are supposed to reduce this volume in their care provision plans. The deviations of the regional targets from the federal ones must be agreed on with the federal Ministry of Health.

*Vertical programs* refer to the national scale programs initiated by the federal government which define activities, federal and regional targets, and the patterns of their joint funding. The main vertical programs are the priority national project “Health” (2006–2012), the preventive care program (2008–present), the healthcare modernization program (2011–2013), the healthcare development program (2018–2024), and the national project “Healthcare” (2019–2024). Vertical programs address specific problems which may not be addressed normally because the lack of resources. Examples are preventive measures, the expansion of care for patients with cardiovascular diseases, cancer, etc. The main source of financing for vertical programs is the federal budget. For example, the share of federal budget in the total amount of public funding for the national project “Healthcare” is 81% (30).

Vertical programs are administered by the federal Ministry of Health and regional health authorities with the major role of the former. The main objective of most vertical programs is to equip state owned providers with new technology and develop advanced specialty (or primary) care. In this way they cementеd tendency to centralized provision and financing of high-tech medical care.

*Pathways of medical care provision* are new type of regulations. Federal law requires the medical care to be provided in agreement with them. Pathways are developed for large groups of diseases and approved by the federal Ministry of Health. As of October 2023, 60 such pathways had been approved. They include:

• pathways of patients receiving medical care in a multi-level system;
• rules for organizing the preventive and curative activities of providers;
• standards for provider equipment and staffing.

Some elements of pathways are compulsory, others, e.g., staffing standards, are non-binding recommendations.

### 3.2 The impact of centralization on changes in the ratio of types of medical care

Main instrument for influencing the structure of service delivery are healthcare utilization targets. The first state guarantee program, approved in 1998, set the target for inpatient care volume 22% lower than the actual one the year before (2.8 and 3.6 bed-days per resident, respectively). The targets for the volume of outpatient care and volume of care in day wards were set higher than the actual volumes. This way the program set the desired vector of structural changes. This line was followed in subsequent years.

At first, this targeting had little effect on volumes of healthcare, because the federal center had insufficient power to enforce regions to follow the targets. Since 2012, the power of the federal Ministry of Health has expanded significantly, new levers of influence on the regions had emerged and regions received new leverage to influence the local service providers. The gap between targets and actual care utilization was reduced (Table 1). Volumes of inpatient care, measured by the number of bed-days per resident, have been steadily declining. For 1998–2019, this indicator decreased by 35%. Hospital bed capacity dropped by 40%. However, in 2019 Russia still retained its international leadership in the number of hospital beds—8.1 beds per 1,000 inhabitants vs. average 4.4 beds in OECD countries (34).

**Table 1:** Target and actual utilization of care in 1998–2019 in Russia.

	1998	2000	2005	2010	2015	2019
Inpatient care (bed days per 1,000 population)^a^						
Target	2,902	2,813	2,813	2,780	2,297	2,180
Actual	3,317	3,298	3,038	2,733	2,393	2,183
Outpatient care (visits per 1,000 population)^a^						
Target	9,198	9,198	9,198	9,500	9,635	9,729
Actual	8,776	9,069	8,523	9,312	8,800	8,393
Day care (patient days per 1,000 population)						
Target	660	749	577	590	675	666
Actual	177	325	457	523	619	636
Emergency care (visits per 1,000 population)						
Target	340	318	318	318	318	300
Actual	346	362	339	336	307	301

Sources: Healthcare in Russia, 2023 (31); The Russian Ministry of Health*,* 2019 (32); The Russian Ministry of Health, 2023 (33).

Note: ^a^The composition of targets have been changing over the period. Targets for different years have been normalized to one comparable indicator.

The reduction in the volume of inpatient care occurred mainly due to the use of reserves accumulated over decades, such as reducing the average duration of hospitalization and downtime of a hospital bed. More complex tools for increasing the efficiency of using a hospital bed (introducing new effective medical and organizational technologies for provision of inpatient care and improving the work of primary health care helping to reduce patients' need for inpatient care) have not received proper application. As to the number of hospitalizations, it even increased slightly (25).

**Figure 1:**
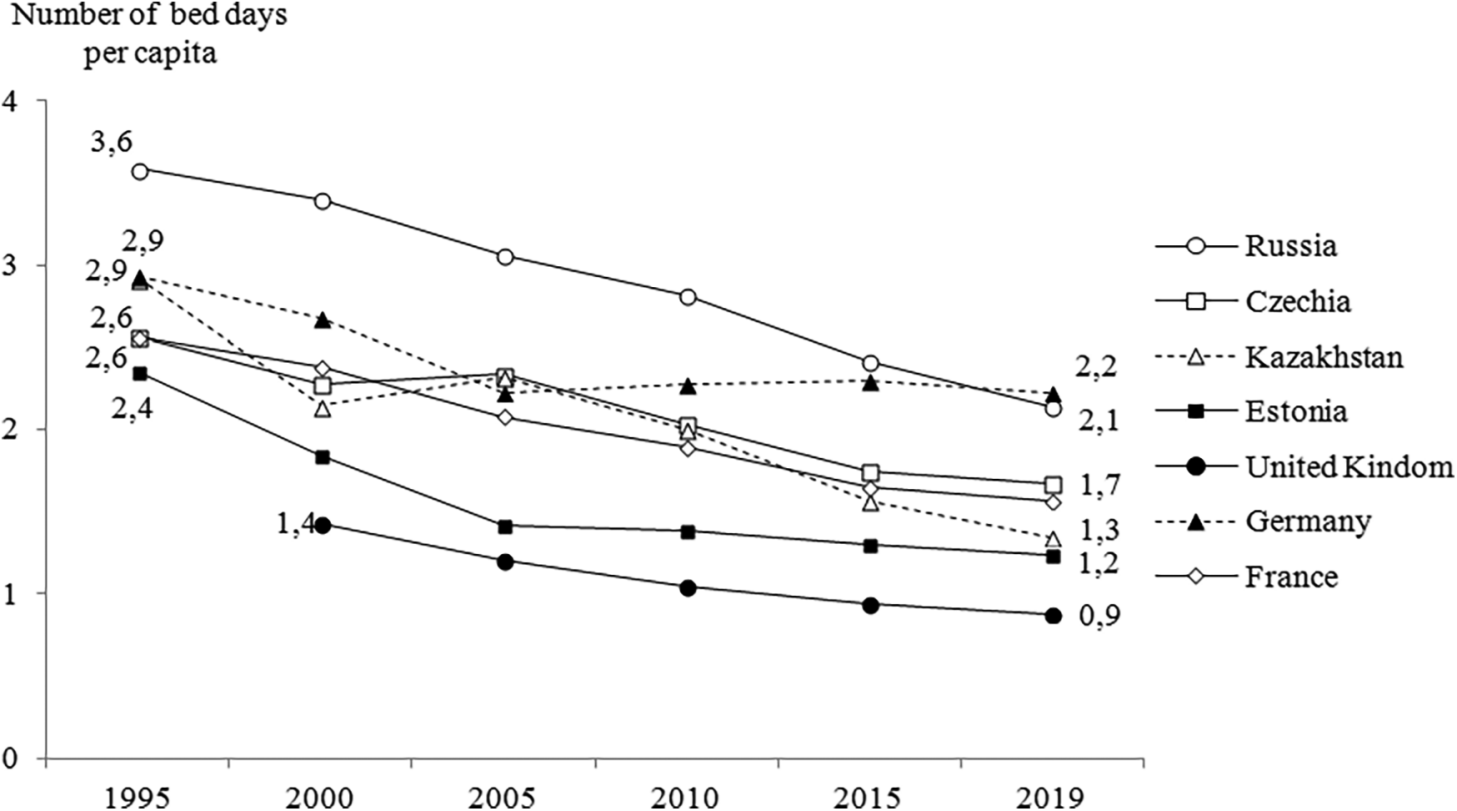
Number of bed days per capita, Russia and selected countries, 1995–2019. Sources: OECD database (22); European Health Information Gateway (23).

Over the past two decades, volume of inpatient care in Russia has declined faster than in European countries, but still remains significantly higher—31% than in the UK, 2.3 times higher than in France. This figure is approximately the same only in comparison with Germany (Figure 1). There is a big gap even with post-Soviet countries—Czechia, Estonia, Kazakhstan (34, 35).

Utilization targets set by PSG have given a strong impetus to the deployment of a network of daycare wards. The number of such beds increased by 230% between 2000 and 2019, the number of patient days per 1,000 residents – by 190%. As a result, the share of patient days in day wards in the sum of total hospital stays increased from 9% to 22.6% (Table 2). This is a clear indication of the substitution of inpatient care for day care.

**Table 2:** Capacity and utilization of day wards in 2000–2019.

	2000	2005	2010	2019	2019
Number of beds in day wards	109 202	198 784	219 690	262 329	255 024
Number of patient days in day wards per 1,000 population	325	457	523	619	636
Number of bed days of inpatient care per 1,000 population	3 298	3 038	2 733	2 393	2 183
The sum of patient days in day wards and bed days of inpatient care per 1,000 population	3 623	3 495	3 256	3 012	2 819
The share of patient days in day wards in the sum of patient days in day wards and bed days of inpatient care per 1,000 population, %	9,0	13,1	16,1	20,6	22,6

Sources: The Russian Ministry of Health, 2019 (32); The Russian Ministry of Health, 2023 (33).

The performance of day wards reflects decisions dictated from the top. Numerous cases of simple medical interventions were transferred from outpatient care to a day ward just to achieve the target. Additional stimulus to use the day care instead ambulatory care was and still is the access to the free drug therapy, not available in outpatient care. As a result, the actual scale of the substitution of hospital care was reduced.

After the first success of the targets’ setting, serious problems have been discovered that complicate the progress of structural reforms. First, the targets do not always take into account the organization of medical care in the regions. Regions are often forced to follow targets without having the real ability to actually reach them due to the specific needs of the local population. Second, utilization targets do not sufficiently take into account the possibilities of service restructuring based on integration of medical care. Centralized solutions set the vector of change in each healthcare sector but cannot ensure effective interaction between them. It is a common situation when the optimization of inpatient care is not supported by the efforts of outpatient clinics, and the comprehensiveness of medical care provided at each stage suffers. Third, the need to follow the decisions of the federal center sometimes forced regions to reduce the capacity or even close medical organizations that are urgently needed by the local populations. Finally, the practice of imitation of structural changes and meeting the targets was widespread, to improve reports and met conditions for additional funding.

### 3.3 The impact of centralization on the organizational structure of service providers

Most healthcare facilities in Russia are owned by state - belongs to federal or regional governments. PHC in urban areas is provided by multispecialty polyclinics—separate for adults and children. Each polyclinic has a catchment area served -by district therapists, district pediatricians, and general practitioners—all of them are referred to as “district physicians”. The catchment population of urban polyclinics ranges from 30,000 to 120,000 people. Large polyclinics employ 15–20 categories of specialists. Hospitals vary in size, the structure of specialties, and the number of beds. The distinction between acute and long-term hospitals does not exist in Russia. Nursing homes, palliative care and post-acute institutions are rare.

The network of Russian medical organizations inherited from the USSR was characterized by a significant number of small providers that were unable to master new medical technologies. E.g., there were hospitals specialized in obstetrics. In the post-Soviet period, the main tendency was the reduction in the number of hospitals and their consolidation. The reduction process accelerated after the transfer of healthcare facilities from the municipal to the regional property in 2006. This made it easier to liquidate small facilities and reduce bed capacity, since municipal authorities could no longer prevent such decisions. Between 2006 and 2012, regional governments reduced the number of hospitals by more than a third. During the same period, there was a maximum reduction in the hospital-bed capacity and in the number of outpatient organizations due to their closure, merger or affiliation with hospitals (25).

A further reduction of the hospital network happened because of the need to find additional funds to increase the remuneration of medical personnel. In 2012, a presidential decree obliged state organizations to increase the remuneration of doctors to the level of 200% of the average wages in the respective region by 2018.The target for nurses was set at 100% (36).

**Figure 2:**
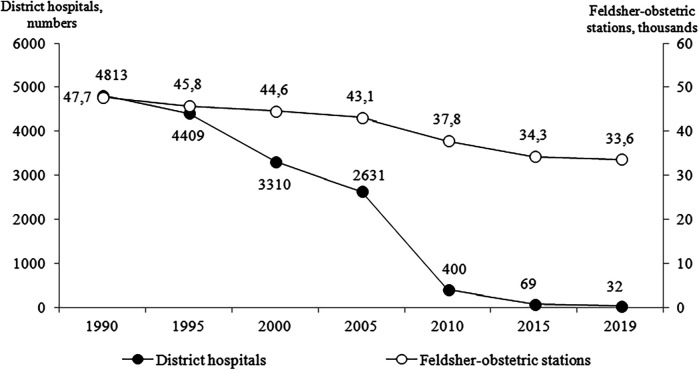
The number of district hospitals (scale on the left) and feldsher-obstetric stations (scale on the right) in 1990-2019. Source: Healthcare in Russia, 2023 (19).

The regions had to find a third of the funds necessary for this salary increase on their own. This led to the simplest unsystematic solution at the regional level—a reduction in the number of physicians in state medical organizations and a further reduction in the network of hospitals. The closure of small hospitals and nursing stations (feldsher-obstetric stations) in rural areas and small towns was especially painful. Their number has been steadily declining (Figure 2), which created a serious tension in the healthcare system.

The reduction in the number of hospitals led to an increase of average capacity of remaining hospitals by 47%—from 160 beds in 1990 to 235 in 2020 (31). This trend is similar to international development. But it is much less driven by the willingness of providers to strengthen their market power. The major motivation for the concentration was to enable stronger management of medical facilities in order to accelerate structural changes in service delivery (37). Among the changes in the organizational structure of service providers, strengthening a multi-level model of service delivery is of particular importance. The centralization of healthcare governance has accelerated this process. The current model includes municipal (district), inter-district, regional and federal levels. They vary in equipment and staffing but are closely linked through a referral system from one level to another, prescribed by the pathways of healthcare provision. The creation of such multi-level model was seen by federal health ministry as a universal solution to increase allocative efficiency. The ministry demanded from regions to create such models everywhere. This model allows resources to be allocated in accordance with the severity/complexity of diseases that should be treated at the prescribed level. It reflects the pattern of population distribution in Russia—a relatively high proportion of the rural population (25%) (38), a low population density in most regions, and large distances between settlements.

**Figure 3:**
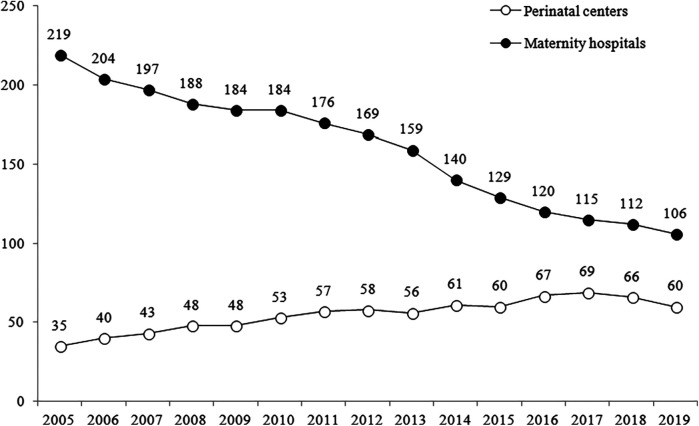
Changes in the number of maternity hospitals and perinatal centers in 2005–2019. Sources: The Russian Ministry of Health, 2002 (27).

New functional structural units have been created in the frames of some federal vertical programs. Centralized governance tends to create new vertical structures. An example is the network of perinatal centers initiated by the federal Ministry of Health for the management of complicated births, premature, and sick newborns (39). The concentration of complicated cases in perinatal centers with qualified personnel, continuing experience and the necessary equipment was logical in the transition to new live birth criteria started in 2012 (40). However, there were not enough qualified personnel to work in the newly established perinatal centers. Therefore, regions had to reduce the number of regular maternity departments and transfer their doctors to the perinatal centers (Figure 3). This led to the transfer of a substantial number of uncomplicated births to perinatal centers. The structure of their activity does not fully correspond to their mission.

As part of the priority national project “Health” (2004–2012) a program of care for myocardial infarction and stroke has been implemented, and a network of regional vascular centers and primary vascular departments (performing less complex interventions for stroke and myocardial infarction) has been established. In 2022, the number of vascular centers reached 215, and primary vascular departments 541. Most of them have been set up on the basis of existing hospitals and their cardiology and stroke departments, that have been heavily re-equipped. This process is ongoing. This program is an unique example of an attempt to quickly and uniformly introduce the use of expensive and efficient technology in a large country. During the implementation of this program, a large lag in the use of thrombolysis for myocardial infarction and ischemic stroke has been largely overcome (41).

The initiator of the creation of vertical models of medical care was not only the federal Ministry of Health, but also some federal clinics. An example of the success is the project of urological care in Voronezh Region in Central Russia. It provides a vertical system of care from PHC to the federal urological center. Inter-district urological centers serve the population of 2–5 municipalities and act as coordinators of patient flows across the stages of service delivery. Integrated pathways have been developed and implemented (42).

### 3.4 The impact of centralization on changes in the structure of activities of healthcare providers

The priority of prevention has traditionally been explicit in Russian healthcare policy. Since 2008, the federal government has implemented a nation-wide vertical program of “dispensarization” (the Program), which is a set of health check-ups and screenings with subsequent secondary prevention interventions for the cases found. This meaning of a term originates from the Semashko model, and is practically unknown in the international literature, where dispensary was a name for the health care setting for the poor (e.g., Boston dispensary) or the unit to distribute the drugs nowadays. The Program uses unified approaches: universal coverage, a single package of services for the early detection of diseases and risk factors, and common performance indicators. All residents over 40 years of age undergo examinations annually, citizens aged 18–39 years old—once every three years. Specialized units have been set up in large polyclinics. The regions were provided with additional funding for the Program, which is administered centrally.

Official data indicate a significant increase in the coverage by check-ups, a high level of disease detection, an increase in the proportion of the population with diseases diagnosed, and an increase in the number of citizens under medical care as a result of screenings (43).

A number of problems emerged, however, caused by the excessive centralization of the Program. The Program includes screening methods, some of which are not evidence based. The uniformity of target population groups and a fixed list of examinations limit the flexibility of regions in responding to local conditions, which dictate the need to provide specific preventive service for some target groups. For example, in a number of northern regions, urolithiasis, thyroid diseases, and hepatitis are especially common, but the detection of these diseases was not provided in the Program. The list of services cannot be modified in accordance with local needs. This list is modified by federal Ministry of Health with general tendency to expansion of screenings.

The Program emphasizes the identification rather than follow-up management of patients. Fulfilling plans to cover the population by testing is a priority for PHC providers, since this indicator is monitored by higher authorities. To nudge physicians to screen all the population in the catchment area, the payment for the Program's activities was extracted from the per capita funding of polyclinics. The decision to start the Program was made at a time when the shortage of PHC doctors had worsened. The Program brought the additional workload. There are many regions in the country where the workload of local physicians exceeds the norm by 100%–150% (instead of mandated 1,700 people in the catchment area of a district physician there are 3,500–4,000) (44).The heavily controlled activities of the Program negatively affected routine medical care with far-reaching consequences for the availability and quality of care. An unexpected consequence is the low level of follow-up of patients with diseases identified due to the Program implementation.

Another example of changes in the functionality of healthcare providers that resulted from centrally made decisions is the introduction of the so-called “New model of primary care organization”. This term is used to denote the reorganization of the work of polyclinics based on lean technologies that should ensure safety and quality, eliminate losses and reduce costs, help create a corporate culture, regulate patient flow, etc. The introduction of this model was initiated by the federal Ministry of Health in 2016, first as a pilot project and then as part of the national “Healthcare” project (45). In 2022, this new model was implemented in 75.1% of polyclinics (43).

Despite the importance of this innovation, paradoxically, it hinders the development of alternative models of PHC such as general practice, integrated medical and social practice, home clinics, and feldsher practice. Unlike in other post-Soviet countries, the institution of independent general practitioners did not exist and not developed in Russia. Only 3% of medical schools provide training in family medicine (46). Some regions, for example, Belgorod region, had initiated their own projects for the development of general practices. But as they did not fit into the content of the corresponding federal projects, the experience of such regions was not replicated nationwide. The multidisciplinary polyclinic with the dominance of specialists remains a dominant model of PHC (47).

Meanwhile, there is an urgent need for a variety of such models. In many regions of the country, there is a large proportion of the population living in remote areas, where it is impossible to provide even a limited range of specialists and there is a need to develop general practices with broad clinical training. But the obsession with unified solutions, resulting from a centralized governance system, poses serious obstacles to their development.

### 3.5 Changes in the way service providers interact

Some initiatives had addressed the interaction between providers. As noted above, a multi-level system of medical care has been created, and pathways for the movement of patients along the system have been prescribed. Digitalization has been developing, aimed at more intensive information exchange between providers. All these areas of integration are supported by the federal government. However, these initiatives are not enough to overcome the fragmentation of healthcare.

In the Russian healthcare system, conceived as an integrated one (large PHC organizations, district physicians responsible for their catchment area, a system of patient referrals in a multi-level system of care), many elements of fragmentation have accumulated. There are insufficient links between individual sectors of the healthcare system, low continuity in the management of patients at different stages of service delivery, insufficient cooperation with social care, etc. A survey of Russian physicians showed in 2020 that the share of district physicians who received information about the hospitalization of all their patients was only 19.6%. The majority of outpatient physicians (72%) do not have regular consultations with hospital doctors on the management of patients after their hospitalization. Only 18% of hospital physicians have access to all electronic medical records. More than half of hospital doctors (57.2%) say their patients are rarely transferred (when needed) to inpatient social care settings, and one in five (20.8%) say such transfers are not practiced at all (48). That is, the centralization of health governance has not contributed much to service integration. And this is because centralization emphasizes vertical connections between different units of healthcare system and prevents the development of horizontal ones.

### 3.6 Impact on the formation of MHI and the purchase of healthcare

The evolution of the MHI system since 1993 has followed the trends of the healthcare governance system, moving from a decentralized to centralized model. In the 1990–2000s, federal rates of budget contributions for MHI of the non-working population, did not exist. Each region determined volume of contribution based on its own budget policy. This led to significant regional differences in the size of these contributions and the share of MHI in public health expenditures. In 2008, the latter was 18%in Khanty-Mansi and 89% in the Republic of Tatarstan (49). Regional healthcare purchasing practices also varied significantly.

The reform of MHI in 2010–2014 was focused on financial and administrative centralization: the accumulation of all MHI contributions in the federal MHI fund, who further allocates them to regional MHI funds; the centralized determination of the rate of MHI contributions for the non-working population in the regions, and unified rules of purchasing medical care (49). The reform was caused by the desire of the federal center to improve the manageability of the MHI system and ensure the financial sustainability of the MHI as a whole and all its regional subsystems.

Centralization had led to a more even distribution of funding but was unable to ensure the equality of regional per capita healthcare expenditure. Rich regions enrich their regional MHI programs with additional funds allocated from their budgets. Per capita healthcare spending in rich regions is two to three times higher than that in the poorest regions (29).

Medical care is purchased under standard contracts with health providers. This makes it difficult to take into account the specifics of a particular provider. Within the framework of a standard contract, it is impossible to determine additional parameters of care (quality, accessibility, etc.) or introduce incentives for technological change. This unified approach reduces the ability of health purchasers to exert managerial influence on providers to improve their performance. Instead of negotiating healthcare parameters declared in the regulation, the current purchasing pattern bears a strong resemblance with directive planning used in the USSR (29).

### 3.7 Impact on the accountability of health authorities

Centralization was followed by strengthening the public upward accountability of regional authorities to the federal government (50). The condition for the region to get MHI funds was the conclusion, since 2015, of an annual agreement between the federal Ministry of Health, the federal MHI Fund and the regional government on the implementation of the PSG. Similar agreements are concluded for the implementation of vertical federal programs. Regional governments are responsible for the use of the financial resources allocated to them and the achievement of the PSG and the vertical programs targets. The federal Ministry of Health has established the performance management system of operating procedures to monitor the regional developments.

As for the social downward accountability of federal and regional authorities to citizens, the modes of informing citizens about their activities have been significantly enriched (50). To a large extent, this was due to the development of information technology. Information about activities began to be posted on the websites of governing bodies, the number of public presentations of the outcomes of health system and plans of health authorities increased. The federal Ministry of Health even established requirements for the content and form of information about the activities of medical organizations posted on the official websites of the Ministry itself, and regional authorities. However, information about the activities of health system is very restricted for public use. This enables health authorities to focus on achievements in their reports, while hiding shortcomings. Feedback from patients, and society as a whole, is poorly expressed (25). Over time, a tendency to limit the content of information posted in the public space began to emerge. Thus, in recent years, the federal Ministry of Health has stopped publishing detailed annual reports on its activities and reports on the implementation of the PSG, replacing them with colorful brief presentations.

### 3.8 Disparities in access to free medical care

Centralization has led to a more even distribution of funding but was unable to ensure the equity of regional per capita public health expenditure. Rich regions supplement their regional MHI programs with additional funds allocated from their budgets. Per capita public health spending in the richest regions is two to three times higher than that in the poorest regions (17). Better funding enables them to invest more in public health and primary care.

**Figure 4:**
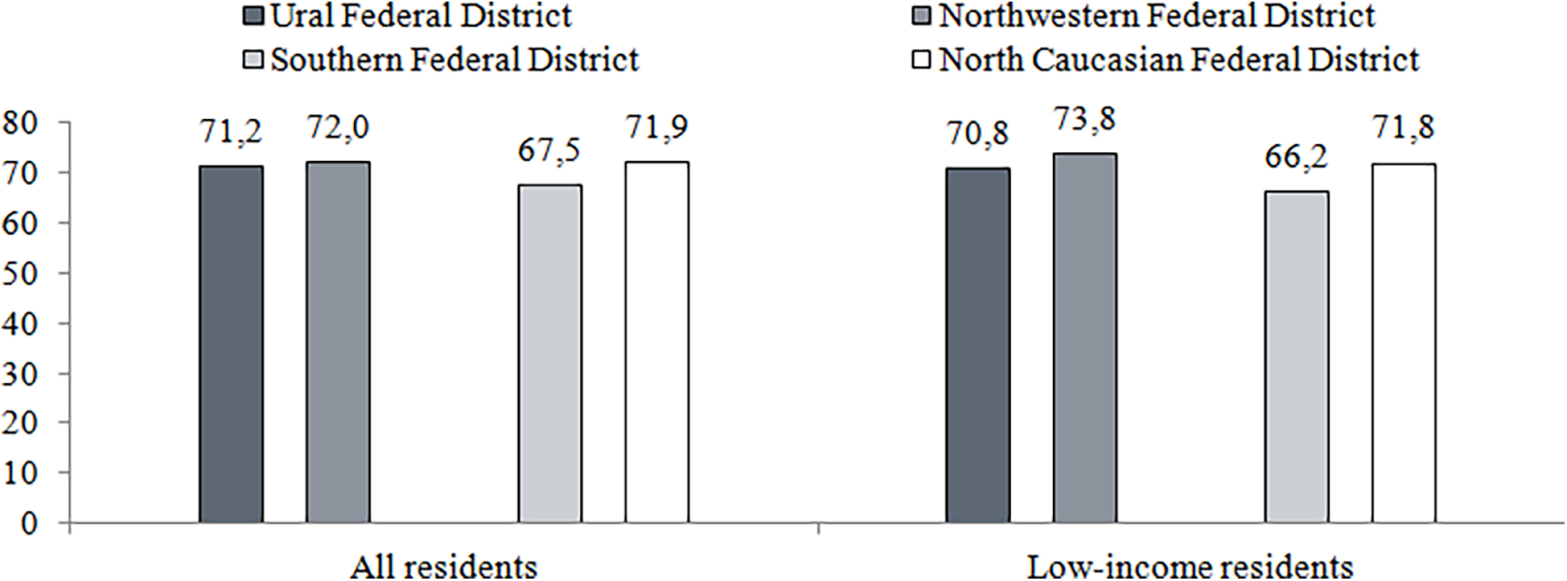
Free medical care seeking, including low-income residents in two richest of federal districts (Ural and northwestern federal districts) and two poorest ones (southern and north Caucasian federal districts), 2002, %. Source: Data of the survey "Readiness for Change" conducted by the National Research University Higher School of Economics in 2022 (https://csils.hse.ru/monitoring_gkp). Calculations of authors.

**Figure 5:**
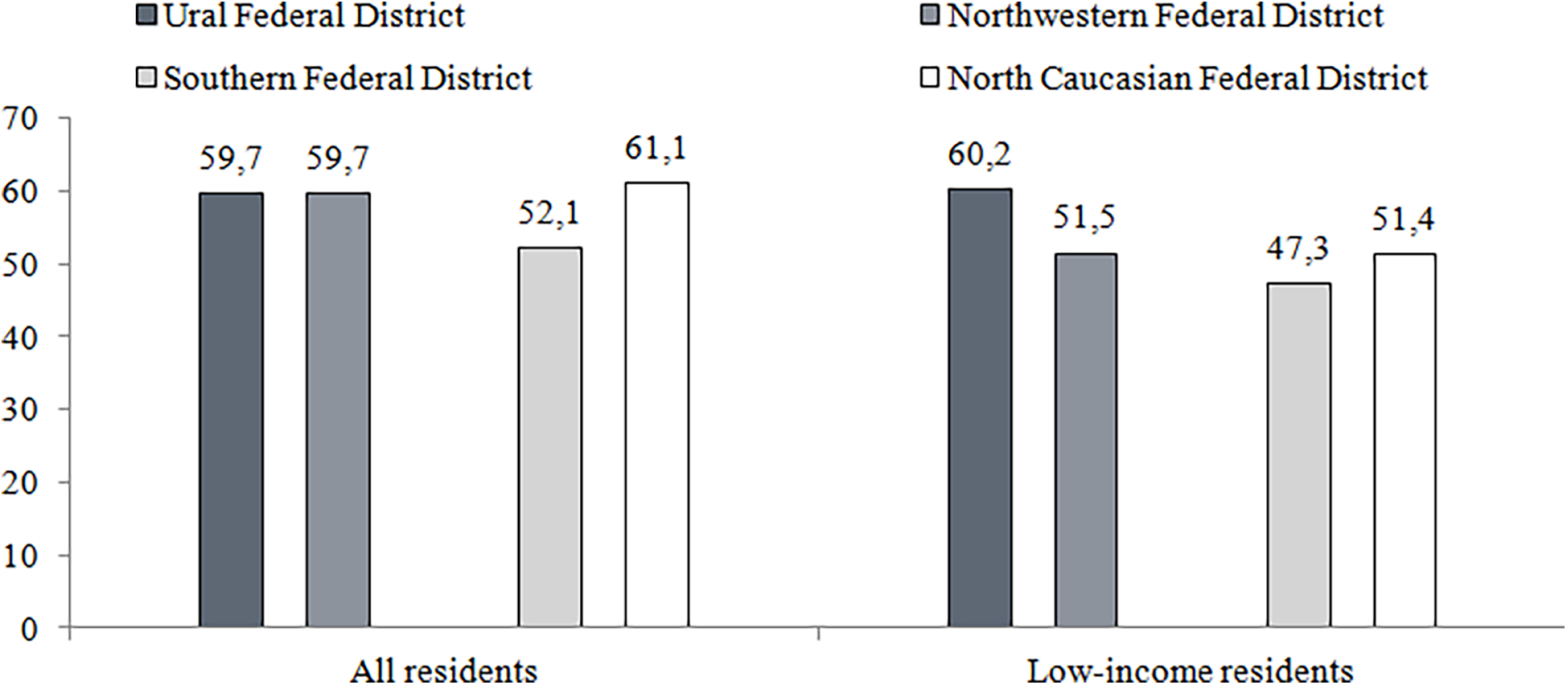
Patient satisfaction with free medical care, including low-income residents in two richest of federal districts (Ural and northwestern federal districts) and two poorest ones (southern and north Caucasian federal districts), 2002, %. Source: Data of the survey "Readiness for Change" conducted by the National Research University Higher School of Economics in 2022 (https://csils.hse.ru/monitoring_gkp). Calculations of authors.

Disparities in the resources of regional health systems affect the accessibility of quality medical care. The survey of 2022 indicates that the share of population contacting health providers varies little for people living in two the richest and two the poorest federal districts (there are 8 federal districts in the country, with 7–16 regions each). However, the level of satisfaction with care varies significantly (Figure 4). The share of respondents who indicated no cases of dissatisfaction is higher in wealthier federal districts. The satisfaction is significantly lower among low-income citizens (Figure 5).

The studies of the regional data indicate that health expenditure is a powerful predictor of the lower mortality (18, 19, 38, 39).

### 3.9 General characteristics of health outcomes

The policy of centralizing healthcare governance has been actively pursued since 2012. According to the annual sociological surveys, the share of adult citizens who were satisfied with the health system in Russia increased from 27% in 2011 to 39% in 2022 (40). The average life expectancy increased from 63.9 to the maximum of 73.4 years in 2023. Since 2012, public funding has stagnated (51), while the age-standardized mortality rate continues to decrease - from 799 to 701 per 100,000 population (women), from 1,636 to 1,351 (men) (25). These positive changes can be partly attributed to the changes in the organization of health care, which have been driven by the improvement of health governance. The centralization has made the system more manageable.

## 4 Discussion

Centralization of healthcare governance after 2000 induced the deep structural changes in the Russian healthcare system.

The major areas of structural reforms driven by centralization of healthcare governance are:

• service delivery structure, including the transfer of a growing part of inpatient care to outpatient settings and day wards;
• institutional organization of health providers, including reduction in the number of hospitals, their consolidation, development of new types of medical organizations (perinatal centers, vascular centers, etc.), and creation multi-level model of service delivery;
• structure of providers activity, including development of prevention;
• changes in the way providers interact (strengthening of providers activity integration in a multi-level system of medical care).

Our findings indicate the major instruments of governance' centralization that have contributed to the structural reforms:

• centralized setting of care utilization targets for service delivery planning;
• introduction of vertical programs in health priority areas;
• designing new organizational forms of service delivery and promoting them throughout the country;
• centrally determined pathways of patients flows in a multi-level healthcare system;
• pooling health finance at the federal level to soften the postcode rationing and to provide resources for restructuring service delivery.

However, centralization brought a number of serious problems that reduce the possibilities of structural reforms. The analysis across major areas of structural reforms indicates that the peculiarity of the Russian approach to centralization is that the federal government not only sets the priorities and directions of structural changes but prescribes specific activities in detail and controls their implementation. These activities are the same for all regions and, accordingly, for all municipalities within each region. This approach has a strong mobilizing value, and works for equality of regions, but it has downsides. Local conditions are not sufficiently taken into account and in a huge country with a variety of socio-economic, climatic, geographical and epidemiological conditions. This is problematic in the implementation of almost all initiatives from the federal center. The “dispensarization” program provides a single set of screening tests, which does not include tests identifying diseases prevalent in individual regions. Federal initiatives to strengthen PHC are focused on the one “new model,” which hinders the development of other models of organization that take into account the peculiarities of population distribution in the country. Decisions to compulsorily increase the salaries of medical workers at the expense of the regions forced them to reduce the capacity of small medical organizations in rural and remote areas, the demand for which is very high.

Centralization, which has advantages in managing the development of the hospital sector, is used mechanically to manage those sectors of healthcare for which consideration of local needs and interaction with the local community are especially important. This mostly relates to PHC. Many health problems that PHC providers deal with, depend from the local working and environmental conditions, living standards, the variety of socially vulnerable groups of the population, etc. At the local level, it is easier to identify the structural changes that are necessary to solve emerging problems, for example, strengthening interaction with social services, local communities, and developing area-specific preventive and curative activities designed for specific population groups. The role of PHC as an integrator of the efforts of various actors in the system at the local level is also important. Rajan et al. makes a point common for the majority of service delivery contexts that “community engagement predominantly takes place within the realm of PHC, as it serves as the convergence point for primary and community care, addressing the holistic health requirements of both individuals and populations” (52). Unlike hospital care, PHC is provided almost entirely at the local level; inter-territorial flows of patients are limited, so centralization of governance is not necessary to build a rational network of PHC providers.

When determining the agenda of structural reforms in the Russian health system, the mechanisms of coordination of federal projects with regional government bodies and professional medical organizations are not sufficiently used. This reduces the validity of decisions made and does not allow the involvement of more stakeholders interested in the implementation of programs and projects. The almost mechanical repetition of federal recommendations in regional documents has become established. Only those activities that are planned by the federal center are executed, regardless of their usefulness.

As a result of the use of uniform solutions, the search for new mechanisms for an aging, financing, and organizing medical care in the regions and at the local level is hampered. Regions are forced to develop only prescribed set of activities and report to the federal center on their implementation. Meeting the targets is a must to receive funding connected to them. Taking on additional tasks in this situation is very difficult. The rare independent innovations that are implemented in some regions are discussed at scientific meetings but are extremely rarely developed to a national scale.

The focus on vertical programs hinders the development of horizontal programs to improve the organization of medical care. Measures to integrate individual parts of the system are poorly developed. It is easier to initiate these activities from the federal center, but their specific content can only be determined locally.

Centralization of healthcare is in the interests of federal center. It wants to maintain the position as the regulator of the healthcare system and the distributor of funds allocated for the development of this system The attitude of regional authorities to the centralization of healthcare is heterogeneous. Rich regions (there are few of them) are interested in weakening centralization, and in particular in weakening the requirements for standardization of medical care, and in transferring to them greater powers in the organization of healthcare, including in carrying out structural changes. Poor regions that depend on subsidies from the federal budget (these are the majority), on the contrary, are ready to give up to the top another part of their powers along with transferring responsibility for selected functions of healthcare system. Municipal authorities would support steps towards decentralization, but with the transfer of resources to them for the development of healthcare. Healthcare providers are interested in weakening universal requirements for standardization of healthcare delivery and would also support steps towards decentralization. Population interests in relation to health governance and structural changes are not clearly expressed. For citizens, the main interest is not to worsen the accessibility of medical services.

In many developed countries, the role of the central government in decision-making on healthcare reforms, including structural changes in the system, is increasing. A study of health policies in 31 OECD countries found that 53% of the reforms were categorized as a central government legislated reform, 23% were non-legislative central government policies (sometimes legally binding) (53). Many countries are, however, looking for mechanisms to strengthen the interest of regional and local governments in the implementation of centrally made decisions. The interaction among various government bodies in adopting the reform agenda is of particular importance. For example, in Germany there is the Health Goals Forum (gesundheitsziele.de) which is a joint coordination initiative by the federal and state governments and over 140 other institutions. It aims to build consensus on national health targets. The National Cancer Control Plan was initiated by the German Ministry of Health but builds upon cooperation between various stakeholders (54).

In Denmark, the centralization of hospital management has given rise to the special mechanism of interaction—binding agreements between different levels of government to ensure coordinated planning of care provided to patients after their discharge from hospital. Along with the centralization of hospital management, measures are being taken to expand the powers of communities in primary and social care. In addition to the structural effect, this increases the involvement of local governments in the implementation of central government initiatives (55).

The potential for centralization is largely determined by the context of the country, including its size. For small countries, the potentially negative aspects of centralization are offset by the relative homogeneity of the territories. For example, the centralization of hospital management in Finland has led to the consolidation of hospitals and the creation of inter-territorial centers. Even if this is accompanied by the closure of a number of local hospitals, the availability of care is not reduced in practice, because the average hospital district has only 12,000 people. The centralization of governance in such a country is less conflicting because the interests of individual municipalities can be harmonized. In Russia, centralized decisions on structural reforms inevitably face different conditions for their implementation. The degree of interest of local communities in hospital reform varies greatly, depending on the distance from inter-territorial centers. The number of losers is sometimes greater than the number of beneficiaries.

Centralization often reflects the desire of the central government to concentrate power. The interest of health care system is the orientation of centralization towards solving specific healthcare management problems, for example, creating a more rational network of hospitals, developing inter-territorial centers, or taking advantage of centralized procurement. With this formulation of the problem, the required degree of centralization becomes apparent. Some problems can be solved with greater involvement of local authorities if they are endowed with the appropriate powers.

The further strengthening of centralization of healthcare governance in Russia is associated with the risks of weakening the effects of government programs and projects. The level of centralization already achieved resulted in insufficient consideration of regional and local conditions. Resolving this requires finding a new balance in the distribution of powers and responsibilities between administrative levels, in a new configuration of the vectors of centralization and decentralization.

A methodologically sound approach is that power-sharing decisions need to be reviewed and adjusted over time to ensure that the governance structure adequately responds to changing needs and policy objectives. In the study of the intergovernmental governance for health, Greer clearly identifies this option: “moving power up and down geographical or organizational levels may be one way of addressing these challenges, but every country (centralized or decentralized) can point to areas where this is done well” (56). In other words, the differentiation of the governance structure in various areas of the healthcare system may foster structural changes without a one-way centralization of power. In the context of Russia, the first step is to empower local governments to organize PHC and create institutional mechanisms for such governance. Their major elements are the authority and the accountability of local governments in this area in an integrated complex with community care.

The major lesson learned is that the centralization of healthcare governance can contribute to structural reforms when the following major conditions are met:

(a) the engagement of regions, communities, and professionals in developing the agenda for reforms,
(b) a special focus on the mechanisms to adapt centralized decisions to the regional and local needs,
(c) interaction among different levels of healthcare governance in the implementation of structural changes,
(d) avoiding centralization of primary health care governance.

## 5 Conclusion

Centralization of healthcare governance has contributed to restructuring the Russian health system, providing a number of positive changes in the ratio of volumes of the health sectors, the organizational structure of service providers, and the structure of their activities. However, centralization has not been accompanied by the use of effective mechanisms of interaction among different levels of government in shaping the structural reforms and their regional implementation. This limits the ability to take into account local conditions. Some uniform solutions are being implemented in regions with negative consequences for the accessibility of medical care locally. An overly centralized model of disease/risk factor screening does not adequately take into account territorial variation in risk groups and does not ensure a balanced relationship between disease detection and subsequent treatment. The new PHC model initiated from the top offers virtually no alternatives. The contradictory impact of centralization requires development of a new balance in the division of powers between different levels of government.

## Data Availability

Publicly available datasets were analyzed in this study. This data can be found here: OECD database, available at: OECD Data Explorer - Hospital aggregates, European Health Information Gateway, WHO Europe, available at: https://gateway.euro.who.int/en/indicators/hfa_540-6100-average-length-of-stay-all-hospitals/#id=19635.
